# Are NORs Always Located on Homeologous Chromosomes? A FISH Investigation with rDNA and Whole Chromosome Probes in *Gymnotus* Fishes (Gymnotiformes)

**DOI:** 10.1371/journal.pone.0055608

**Published:** 2013-02-06

**Authors:** Susana S. R. Milhomem, Priscilla C. Scacchetti, Julio C. Pieczarka, Malcolm A. Ferguson-Smith, José C. Pansonato-Alves, Patricia C. M. O’Brien, Fausto Foresti, Cleusa Y. Nagamachi

**Affiliations:** 1 Laboratório de Citogenética, Instituto de Ciências Biológicas, Universidade Federal do Pará, Belém, Pará, Brazil; 2 Instituto de Biociências de Botucatu, Departamento de Morfologia, Universidade Estadual Paulista, Botucatu, São Paulo, Brazil; 3 Cambridge Resource Centre for Comparative Genomics, Department of Veterinary Medicine, University of Cambridge, Cambridge, United Kingdom; University of Florence, Italy

## Abstract

*Gymnotus* (Gymnotiformes, Gymnotidae) is the most diverse known Neotropical electric knife fish genus. Cytogenetic studies in *Gymnotus* demonstrate a huge karyotypic diversity for this genus, with diploid numbers ranging from 34 to 54. The NOR are also variable in this genus, with both single and multiple NORs described. A common interpretation is that the single NOR pair is a primitive trait while multiple NORs are derivative. However this hypothesis has never been fully tested. In this report we checked if the NOR-bearing chromosome and the rDNA site are homeologous in different species of the genus *Gymnotus*: *G*. *carapo* (2n = 40, 42, 54), *G*. *mamiraua* (2n = 54), *G*. *arapaima* (2n = 44), *G*. *sylvius* (2n = 40), *G*. *inaequilabiatus* (2n = 54) and *G*. *capanema* (2n = 34), from the monophyletic group *G*. *carapo* (Gymnotidae-Gymnotiformes), as well as *G*. *jonasi* (2n = 52), belonging to the G1 group. They were analyzed with Fluorescence *in situ* hybridization (FISH) using 18S rDNA and whole chromosome probes of the NOR-bearing chromosome 20 (GCA20) of *G*. *carapo* (cytotype 2n = 42), obtained by Fluorescence Activated Cell Sorting. All species of the monophyletic *G*. *carapo* group show the NOR in the same single pair, confirmed by hybridization with CGA20 whole chromosome probe. In *G*. *jonasi* the NORs are multiple, and located on pairs 9, 10 and 11. In *G*. *jonasi* the GCA20 chromosome probe paints the distal half of the long arm of pair 7, which is not a NOR-bearing chromosome. Thus these rDNA sequences are not always in the homeologous chromosomes in different species thus giving no support to the hypothesis that single NOR pairs are primitive traits while multiple NORs are derived. The separation of groups of species in the genus *Gymnotus* proposed by phylogenies with morphologic and molecular data is supported by our cytogenetic data.

## Introduction

**Figure 1:**
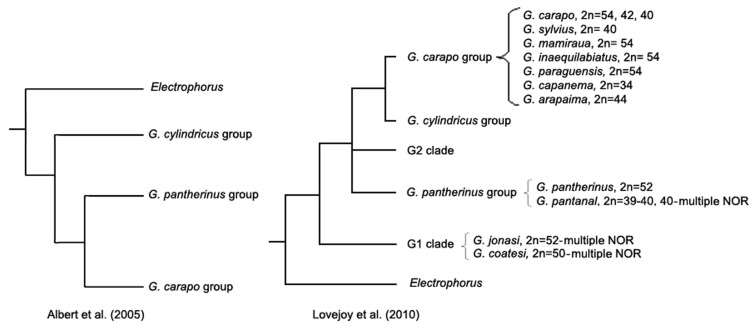
Phylogeny of the genus *Gymnotus* adapted from [3] and [4] with chromosomal information cited in this paper.

*Gymnotus* (Gymnotiformes, Gymnotidae) is the most diverse known Neotropical electric knife fish genus. It currently holds 37 valid described species [1] and is distributed within a large geographic region, from the south of Mexico to the north of Argentina. There are 19 species described in the Amazon region in Brazil [1]. This genus contains three groups: *G*. *carapo*, *G*. *pantherinus* and *G*. *cylindricus* [2]. This subdivision can be found in the phylogeny of Albert et al. [3], where *G*. *cylindricus* is the basal group. However, Lovejoy et al. [4] proposed a new phylogeny with five groups, where the *G*. *pantherinus* group is divided into groups G1, G2 and *G*. *pantherinus*. In the consensus phylogeny, G1 is the basal group while *G. cylindricus* and *G. carapo* group are the more derived condition, with the *G. pantherinus* and G2 groups as sister groups. All studies support the monophyly of the *G*. *carapo* group (Figure 1).

Cytogenetic studies in *Gymnotus* demonstrate a huge karyotypic diversity for this genus. Diploid numbers range from 34 chromosomes in *G*. *capanema* to 54 in *G*. cf. *carapo, G. mamiraua, G. paraguensis* and *G*. *inaequilabiatus.* Variations in the karyotypic formula among species and populations result from differences in chromosome morphology (see [1], [5]). On the other hand, sex-related polymorphisms are found only in *G*. *pantanal*, which has a X_1_X_1_X_2_X_2_/X_1_X_2_Y multiple sex chromosome system [6].

NORs, composed of rRNA genes 5,8S, 18S and 28S, are important markers for evolutionary chromosome studies [7]. Interspecific and intraspecific polymorphism in NORs have been documented in several groups, with variation in the number of NORs per genome, in the chromosomal location of NOR sites, in the relative sizes of individual NORs, and in the number of active NOR sites per cell [8]. These events are commonly seen in fish, where the rDNA loci have been shown to be highly dynamic. For example, three NOR chromosomes, including one W sex chromosome, occur in some species of *Triportheus* [9]; several chromosomes with rDNA are known in *Salmo truta* [10]; similar variation is found in different populations of *Characidium gomesi* [11], and in *Astyanax scabripinnis* [12]. Variations in the number, size and position of rDNA loci on the sex chromosome (varying from two to eight) have also been observed in *Salvelinus alpinus* [13].

In Gymnotiformes polymorphisms are described in the location of the NORs, mainly involving paracentric inversions in the karyotype of the *G*. *carapo* group [14]. However, the presence of a single NOR (one pair) is the most common trait, as found in *Apteronotus albifrons*, *Sternopygus macrurus*, *Eigenmannia* sp, *E*. *virescens*, *Steatogenys elegans, S. duidae*, *Gymnotus carapo*, *G. paraguensis*, *G*. *sylvius, G.* cf. *carapo*, *G. inaequilabiatus*, *G*. *pantherinus*, *G. mamiraua* and *G. arapaima* [1], [5], [15]–[25]. In *Electrophorus electricus* a single NOR was found on a sample from River Araguaia and three NOR-bearing chromosomes were identified in a fish from River Amazonas [26]. On the other hand, in *G*. *pantanal* [27], *G*. *jonasi* [22] and *G. coatesi* [28] the NORs are multiple (more than one pair).

A common evolutionary interpretation of this variation is attributed to Hsu *et al.* [29] who assume that a single NOR pair is a primitive (plesiomorphic) condition while multiple NORs are a derived (apomorphic) trait [16], [30]–[33]. For this hypothesis to be correct two conditions must be achieved: 1) for species with a single NOR the NOR-bearing chromosomes must be homeologous in the different species within the same taxonomic group. Since it is the primitive condition, it must have appeared once and spread over the species. 2) A phylogenetic study should confirm that primitive species have a single NOR pair while derivate species have multiple NORs. The achievement of only one of these conditions is not enough to confirm the hypothesis. With time this old hypothesis became very popular but (or maybe because) until recently there were no tools to test it. Without the precise identification of chromosome homeologies in fish karyotypes, the comparative analysis in different species has been made on the assumption that there is homeology among chromosomes of similar size, morphology and presence of NORs. The absence of compartmentalization in GC or AT rich regions (for revision, see [34]) explains the absence of G-banding in these organisms and the difficulty in obtaining whole chromosome probes by FACS compared to the situation in mammals [35]–[38]. Nagamachi *et al.* [39] demonstrated this by using whole chromosome probes of *Gymnotus carapo* (2n = 42). The chromosomes were separated in groups of pairs with similar size. Only the NOR-bearing chromosome (pair GCA20) was individually separated because of its large amount of repetitive sequences.

**Figure 2:**
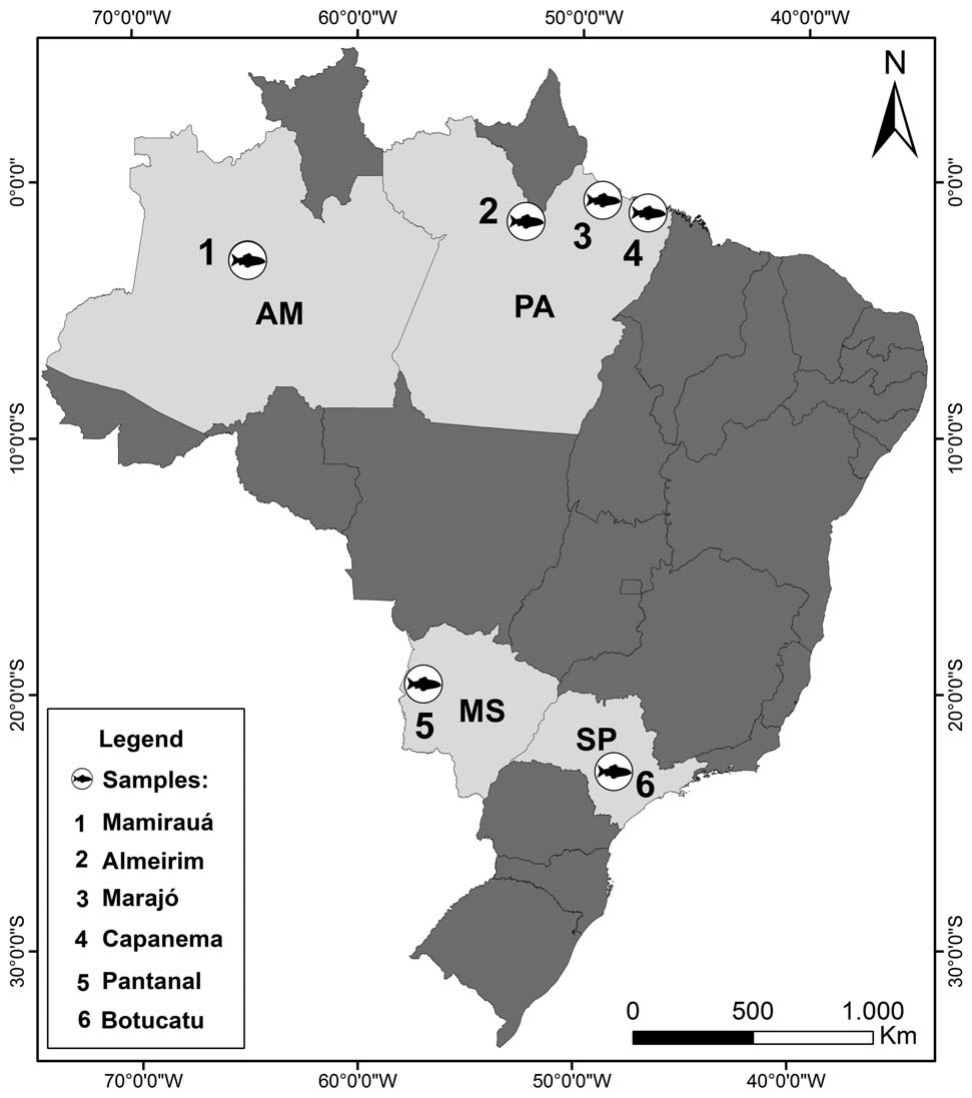
A map showing the places where the samples were collected.

In the present study we check the homeology of the association, sites of rDNA with the NOR-bearer chromosome, by doing dual-color FISH with 18S rDNA and whole NOR-bearer chromosome probes (GCA 20, [39]) on different species of *Gymnotus* from different places in Brazil (Figure 2). Also, we analyzed the results on a phylogenetic perspective, to test the hypothesis that the species with single NOR pairs are primitive. We give results for *G*. *carapo* (GCA, 2n = 40, 42 and 54), *G*. *mamiraua* (GMA, 2n = 54), *G*. *arapaima* (GAR, 2n = 44), *G*. *sylvius* (GSY, 2n = 40), *G*. *inaequilabiatus* (GIN, 2n = 54), *G*. *capanema* (GCP, 2n = 34) and *G*. *jonasi* (GJO, 2n = 52). The results of FISH using only 18S rDNA probes were already reported for most of the species [1], [5], [25], apart from the samples from Almeirim and Marajo Island which are new results (Table 1, Figure 2).

**Table 1:** Some information on the species analyzed in this work.

Species	Coordinates	Locality	2n	KF
*G*. *mamiraua* (GMA)	03°01′41.8″S/064°51′16.6″W	Mamirauá-AM	54	46m/sm+8st/a
*G*. *carapo* (GCA)	00°42′03.2S/049°10′42.1W	Marajó-PA	42	30m/sm+12st/a
*G*. *carapo* (GCA)	01° 31′ 34.2″S/052°33′37.9″W	Almeirim-PA	40	34m/sm+6st/a
*G*. cf. *carapo* (GCA)	19°34′34″S/57°02′17″W	Pantanal-MS	54	38m/+12sm+/4st
*G*. *arapaima* (GAR)	03°02′49.1″S/064°51′02.2″W	Mamirauá-AM	44	26m/sm+18st/a
*G*. *capanema* (GCP)	01°11′45″S/47°10′51″W	Capanema-PA	34	20m/sm+14st/a
*G*. *jonasi* (GJO)	03° 02′49.1″S/064° 51′ 02.2″W	Mamirauá-AM	52	52 (12 m-sm+40st-a)
*G*. *sylvius* (GSY)	22° 59′25″S/48°25′40″W	Botucatu-SP	40	22m/+12sm/+6st
*G*. *inaequilabiatus* (GIN)	22° 59′25″S/48°25′40″W	Botucatu-SP	54	42m/+10sm/+2a

2n = diploid number and KF = karyotypic formula.

## Results

FISH with 18S rDNA probe demonstrate the location of the NOR sites confirming previous publications that these occur in the same positions revealed by Ag-NO_3_ staining in the species GCA, GMA, GAR, GSY, GIN and GCP. New information is given for GCA samples from Almeirim and Marajo Island (Table 1, Figure 2). In GJO hybridization is found in three acrocentric pairs (two pairs with interstitial signals in the long arm and one pair in the short arm).

**Figure 3:**
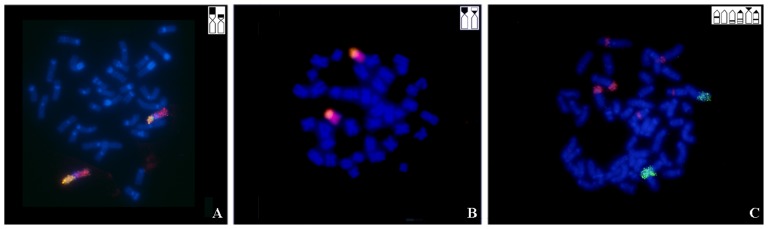
18S rDNA probe FISH (green) and whole NOR-bearing chromosome probe FISH (red) in A) *G. capanema*; B) *G. sylvius* and C) *G. jonasi*. The ideogram on each image shows the position of NOR.

In all the species studied, chromosome painting with the whole NOR-bearing chromosome probe (GCA20) produced a signal in just one chromosome pair. The results with dual color FISH demonstrate the association sites of the rDNA with NOR-bearing chromosome in the species of *Gymnotus carapo* group, that has single NOR. Figure 3 shows examples of this for the species *G. capanema* (Fig. 3A) and *G. sylvius* (Fig. 3B). This result was found on the other species with exception of GJO, where the hybridization is in the distal half of the long arm of a large acrocentric chromosome, pair 7, which is not one of the NOR-bearing chromosomes (Fig. 3C).

## Discussion

In the present work we have used FISH to map NOR numbers and locations on a (partly resolved) *Gymnotus* species tree and characterized the autosome that bears it.

Using the rDNA probe we confirmed that, with the exception of GJO, which has rDNA sequences in three chromosome pairs, all the species have a NOR in just one chromosome pair. The multiple NOR trait has been found only in two other species previously: *G. pantanal* (GPN) that has rDNA in three chromosomes [27] and *G. coatesi* (GCO) that has at least 7 pairs of NOR-bearing chromosomes [28]. There is the possibility that these species can be polymorphic for the number of NORs. However, the genus *Gymnotus* has been studied by others and us in many papers in recent years. In reference [17] we studied 10 *G. carapo* with 2n = 42 and 7 with 2n = 40. In reference [22] we analyzed 12 *G. arapaima*, 23 *G. mamiraua*, 2 *G. jonasi* and 9 *G. capanema*; in reference [5] it were analyzed 23 *G. sylvius*, 18 *G. inaquelabiatus*, 3 *G. pantherinus* and 3 *G. c/carapo*. There are no NOR polymorphism on any of theses samples. So we believe that we can rule out this possibility.

The results of dual color FISH with the 18S rDNA and the whole NOR-bearing chromosome probe, GCA20, demonstrate painting in only one chromosome pair in all the species analyzed, with conserved synteny among different karyotypes. In the *G. carapo* group, the hybridization of this probe extended all over the NOR-bearing chromosome (Figure 3A, 3B), showing that the chromosome/rDNA locus association is conserved in this group of species. In GJO this association was not found (Figure 3C), since the probe of GCA20 painted half a large acrocentric pair (pair 7), which is not one of the NOR-bearing chromosomes (pairs 9, 10 and 11). These results show that the karyotype of GJO has a different organization of rDNA sites and that the GCA20 is associated with another chromosome in a translocation or in a *tandem* fusion rearrangement.

If we accept the phylogeny proposed by Lovejoy et al. [4], where the group *G. carapo* is the most derived and group G1 is the most basal, we may conclude that the single NOR with the conserved chromosome/rDNA locus association shared by the *G. carapo* group is a synapomorphy for the group. *Gymnotus pantherinus* also has a single NOR chromosome, similar to the one in the *G. carapo* group. In GPN there are three NOR-bearing chromosomes (one pair and an additional chromosome from another pair), where one is similar to that found in the *G. carapo* group. Although GPN has not being analyzed by Lovejoy et al. [4], it probably belongs to the group G2 as it is a sister species of *G. anguillaris* [27]. If GPA and GPN share the chromosome/rDNA locus association, this association is likely to have happened before the appearance of the *G. carapo* group.

In the classification of Albert et al. [3] all species belong to the group *G. pantherinus*, except *G. cylindricus* and the *G. carapo* group. In the alternative classification [4] the *G. pantherinus* group is split into three monophyletic groups. Our data show that, while the *G. carapo* group has homogeneity of NORs, the *G. pantherinus* group is heterogeneous. Species GPA has a NOR similar to the *G. carapo* group, while multiple NORs are found in GJO ([22], present study) and GCO [28], apart from GPN [27], already mentioned. Besides this, our studies demonstrate that GJO and GCO have multiple NORs with different patterns of distribution of rDNA loci, involving different chromosomes. The same can be said about the “single NOR” trait, for *Sternopygus* also has a single NOR, but the chromosome involved is different from that in the *G. carapo* group (data not shown), as well as in *Electrophorus* [26]. So it cannot be claimed *a priori* that the single or multiple NOR conditions can be primitive or derived *per se*, since each of these conditions in one species can have an origin and composition very divergent from the one found in other species.

The NOR heterogeneity observed in the *G. pantherinus* group of Albert et al. [3], in contradiction with the homogeneity of the *G. carapo* group, suggests that the *G. pantherinus* group is composed of more than one monophyletic group, supporting the phylogeny of Lovejoy et al. [4]. Actually, Albert et al. [4] included many species in the *G. pantherinus* group because it was not possible to define precisely their phylogenetic relationships using only morphology data. Anyway, these results show clearly the huge mobility of the rDNA cistrons.

Additional studies, with detailed analysis using whole chromosome and rDNA probes in species from different families of Gymnotiformes, will permit more precise conclusions on the differentiation and distribution of NORs in this order. The study of GJO using FISH with other whole chromosome probes as described in Nagamachi et al. [39] will allow an improved understanding of the amount of chromosomal divergence between these species.

## Materials and Methods

Samples were collected in rivers from the Amazon basin (Pará and Amazonas states), Paranapanema basin (São Paulo state) and Paraguay basin (Mato Grosso do Sul state) (Table 1, Figure 2). Some of these specimens were previously studied [1], [5], [17], [20], [21]. Samples were deposited at the Museu de Ciências e Tecnologia da Pontifícia Universidade Católica do Rio Grande do Sul (GCA from Marajó), at Museu Paraense Emílio Goeldi (GCA from Almeirim and GCP from Capanema), at Museu da Reserva de Desenvolvimento Sustentável Mamirauá (GMA, GAR and GJO from Mamirauá) and at the fish collection from the Laboratório de Biologia e Genética de Peixes (LBP) from UNESP from Botucatu, vouchers 11160 (GSY), 9836 (*G*. cf. *carapo*) and 11154 (GIN).

The chromosome preparations were obtained following [40]. The Ag-NO3 staining followed [41]. The FISH with the 18S rDNA probe from *Prochilodus argenteus* was made according to [42]. The whole chromosome probe of the NOR-bearing chromosome (GCA20) of *G*. *carapo* (cytotype 2n = 42) was obtained by Fluorescence Activated Cell Sorting [39]. Dual color FISH was made using the (GCA20) probe, labeled with dUTP-Cy3, and the 18S rDNA probe, labeled with Digoxigenin and detected with Anti-Digoxigenin-FITC. The detection followed [43], with modifications [39]. The images were captured using a CCD Axiocam Mrm camera connected to a Zeiss Axiophot microscope and Axiovision 3.0 software, where the images were pseudocolored. Brightness and contrast levels were adjusted using Adobe Photoshop 7.1.

### Ethics Statement

This study was analyzed by the Ethics Committee on Animal Research from the UNESP University and got the authorization number 395-CEUA. JCP has a Permanent License for Collecting Zoological Samples in Brazil, Permit Number 13248, ICMBio (Chico Mendes Institute for the Conservation of Biodiversity, Brazilian Ministry for Environment).
